# AIDHE: A Framework for Advancing Age-Inclusive Research Practices

**DOI:** 10.1093/geroni/igaf122.136

**Published:** 2025-12-31

**Authors:** Susan Whitbourne

**Affiliations:** University of Massachusetts Boston, Boston, Massachusetts, United States

## Abstract

The AIDHE model views age inclusivity as incorporating seven domains that reflect all activities within institutions of higher education. Central to the mission of many universities, the domain of research includes such age-inclusive practices by research offices as stimulating new investigations on aging through workshops, small grants, featured newsletter stories, support of travel, and assistance in grant writing. The present paper will summarize the AIDHE model in the domain of research and describe the results of the national survey’s findings on the presence of age-inclusive practices and awareness of these practices among the 23 participating institutions. Administrators completed the ICCS Inventory, indicating which age-inclusive research practices their offices supported and also enumerated how many age-related funding agencies supported sponsored research on their campuses. Across all institutions, such age-inclusive practices were reported in only 61%, making research the second-lowest of all domains included in the inventory. Climate survey data from faculty on items tapping awareness of age-inclusive practices revealed even lower awareness, such as in the area of stimulating research discussions on aging, with 39% acknowledging whether these discussions occur on campus. These findings will be discussed along with suggestions for strategies that institutions can embark on to support and encourage broader awareness of the need for research on aging, both in basic and applied areas.

